# Comparison of the Therapeutic Effects of [^211^At]NaAt and [^131^I]NaI in an NIS-Expressing Thyroid Cancer Mouse Model

**DOI:** 10.3390/ijms23169434

**Published:** 2022-08-21

**Authors:** Tadashi Watabe, Yuwei Liu, Kazuko Kaneda-Nakashima, Tatsuhiko Sato, Yoshifumi Shirakami, Kazuhiro Ooe, Atsushi Toyoshima, Eku Shimosegawa, Yang Wang, Hiromitsu Haba, Takashi Nakano, Atsushi Shinohara, Jun Hatazawa

**Affiliations:** 1 Department of Nuclear Medicine and Tracer Kinetics, Osaka University Graduate School of Medicine, Suita 565-0871, Japan; 2 Institute for Radiation Sciences, Osaka University, Suita 565-0871, Japan; 3 Core for Medicine and Science Collaborative Research and Education, Project Research Center for Fundamental Sciences, Osaka University Graduate School of Science, Suita 565-0871, Japan; 4 Nuclear Science and Engineering Center, Japan Atomic Energy Agency, Shirakata 2-4, Tokai 319-1195, Japan; 5 Research Center for Nuclear Physics, Osaka University, Suita 567-0047, Japan; 6 Department of Molecular Imaging in Medicine, Osaka University Graduate School of Medicine, Suita 565-0871, Japan; 7 Nishina Center for Accelerator-Based Science, RIKEN, Wako 351-0198, Japan; 8 Department of Chemistry, Graduate School of Science, Osaka University, Toyonaka 560-0043, Japan

**Keywords:** [^211^At]NaAt therapy, [^131^I]NaI therapy, thyroid cancer, multiple administration, alpha therapy

## Abstract

Astatine (^211^At) is an alpha-emitter with a better treatment efficacy against differentiated thyroid cancer compared with iodine (^131^I), a conventional beta-emitter. However, its therapeutic comparison has not been fully evaluated. In this study, we compared the therapeutic effect between [^211^At]NaAt and [^131^I]NaI. In vitro analysis of a double-stranded DNA break (DSB) and colony formation assay were performed using K1-NIS cells. The therapeutic effect was compared using K1-NIS xenograft mice administered with [^211^At]NaAt (0.4 MBq (n = 7), 0.8 MBq (n = 9), and 1.2 MBq (n = 4)), and [^131^I]NaI (1 MBq (n = 4), 3 MBq (n = 4), and 8 MBq (n = 4)). The [^211^At]NaAt induced higher numbers of DSBs and had a more reduced colony formation than [^131^I]NaI. In K1-NIS mice, dose-dependent therapeutic effects were observed in both [^211^At]NaAt and [^131^I]NaI. In [^211^At]NaAt, a stronger tumour-growth suppression was observed, while tumour regrowth was not observed until 18, 25, and 46 days after injection of 0.4, 0.8, and 1.2 MBq of [^211^At]NaAt, respectively. While in [^131^I]NaI, this was observed within 12 days after injection (1, 3, and 8 MBq). The superior therapeutic effect of [^211^At]NaAt suggests the promising clinical applicability of targeted alpha therapy using [^211^At]NaAt in patients with differentiated thyroid cancer refractory to standard [^131^I]NaI treatment.

## 1. Introduction

Radioactive iodine (RAI) therapy using [^131^I]NaI has a long history of use in patients with differentiated thyroid cancer after thyroidectomy for the ablation of thyroid tissue remnants, neoadjuvant therapy, and the treatment of metastatic lesions [1,2,3]. However, some patients do not achieve sufficient therapeutic effects with RAI therapy, even with sufficient radioiodine uptake [3,4]. For these patients, a more effective therapy using an alpha emitter targeting the sodium/iodide symporter (NIS) can be considered.

In recent decades, Astatine (^211^At) has garnered attention as a halogen isotope, with chemical properties similar to those of iodine such as anion formation, but it emits alpha particles that have higher linear energy transfer (LET) [5,6,7]. Previously, ^211^At was labelled with a monoclonal antibody for the clinical treatment of brain tumour via intracavity injection and ovarian cancer via intraperitoneal infusion [8,9]. Intravenous administration of ^211^At has not been performed in the clinical setting as an antitumour treatment, except for the preparation for stem cell transplantation (ClinicalTrials.gov Identifiers: NCT03128034, NCT03670966, and NCT04083183).

Petrich et al. reported that ^211^At accumulated in NIS-expressing organs and that the administration of ^211^At suppressed the growth of differentiated thyroid cancer xenografts [10]. In our previous study, we found that the uptake of ^211^At in K1-NIS cells was enhanced using ^211^At solution treated with ascorbic acid and demonstrated the dose-dependent tumour-suppressive effect of [^211^At]NaAt solution in NIS-expressing thyroid cancer models [11]. In addition, in our toxicity study, we found that a single intravenous administration of [^211^At]NaAt solution (up to 50 MBq/kg) showed no severe adverse effects in normal mice, suggesting that [^211^At]NaAt solution could be used as an ideal therapy for iodine-avid differentiated thyroid cancer instead of RAI therapy [12,13]. However, we only performed a simple in vitro cellular survival assay using K1-NIS cells treated with [^211^At]NaAt and [^131^I]NaI solution [11]. A detailed comparison of [^211^At]NaAt and [^131^I]NaI, including therapeutic effects in vivo, has not been performed. In the present study, we compared the therapeutic effects of [^131^I]NaI and [^211^At]NaAt with single and multiple administrations, as well as performed in vitro analysis of DSBs of deoxyribonucleic acid (DNA) and colony formation assays with absorbed dose estimation.

## 2. Results

### 2.1. Observation of DSBs of DNA and Colony Formation in K1-NIS cells

**Figure 1:**
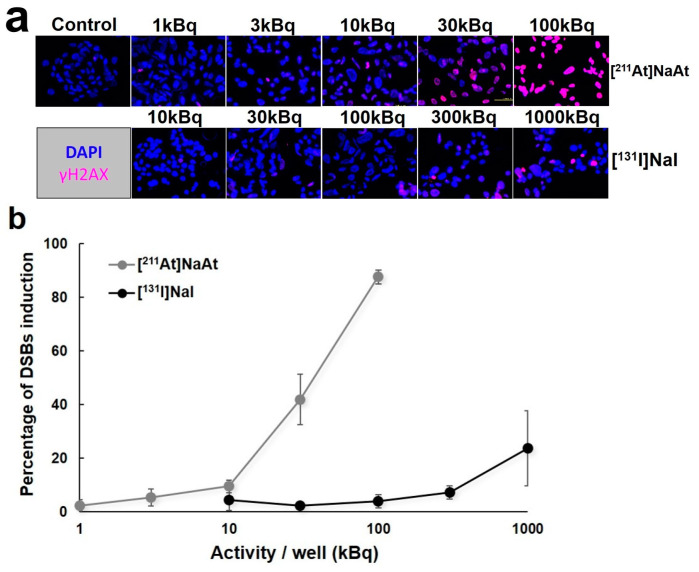

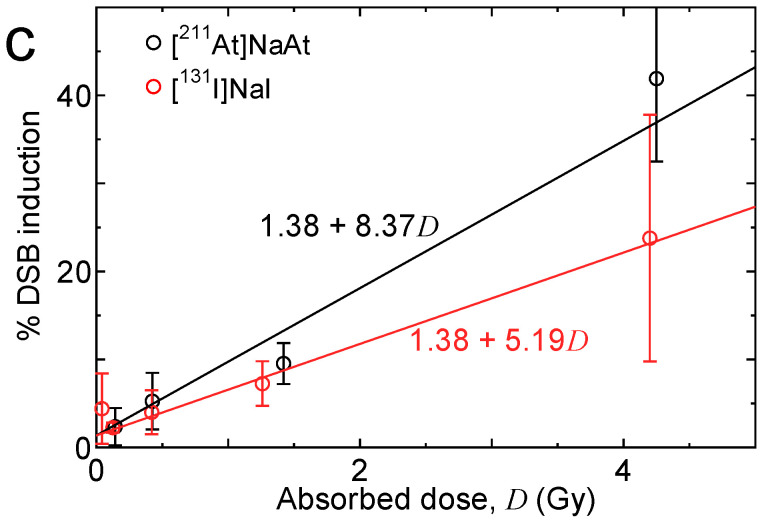
(**a**) Stained images of K1-NIS cells treated with the [^211^At]NaAt solution and [^131^I]NaI solution. Cell nuclei were stained blue and double-stranded break (DSB) induction signals were stained pink. (**b**) The percentage of DSB induction by [^211^At]NaAt and [^131^I]NaI. Percentage of DSB induction (DSBs%) was calculated using the formula: area of DNA damage (pink area)/nuclear morphology (pink area + blue area) × 100. (**c**) Relationship between %DSB induction and absorbed dose. The results of linear fitting of the experimental data are also shown in the figure.

**Figure 2:**
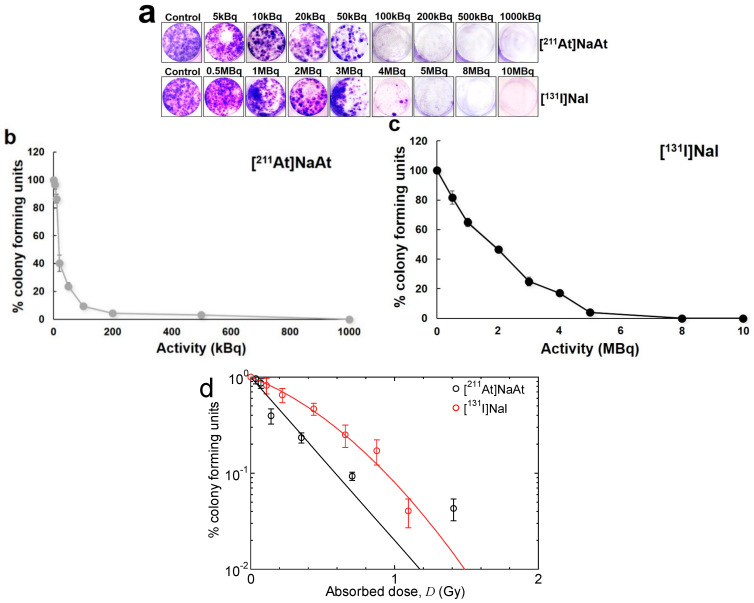
(**a**) K1-NIS cell images stained with crystal violet and (**b**,**c**) % colony-forming units of K1-NIS cells treated with [^211^At]NaAt and [^131^I]NaI solutions. (**d**) Relationship between % colony-forming units and absorbed dose. The results of the linear and linear–quadratic fittings of [^211^At]NaAt and [^131^I]NaI data, respectively, are also shown in the figure. For [^211^At]NaAt, the experimental data for [^211^At]NaAt with the absorbed doses above 1 Gy were excluded from the fitting, because they were probably influenced by the heterogeneity of the radioactive concentration in each cell.

The results of DSB induction are shown in Figure 1. The [^211^At]NaAt caused a higher number of DSBs than [^131^I]NaI at 20 min after irradiation, and their relative biological effectiveness (RBE) was 1.61. The results of the colony formation assay using K1-NIS cells after irradiation with [^131^I]NaI and [^211^At]NaAt solutions are shown in Figure 2. The RBE for 10% colony-forming units estimated from the fitting results was approximately 1.60.

### 2.2. Biodistribution of [^131^I]NaI and [^211^At]NaAt in K1-NIS Xenograft Mice

**Figure 3:**
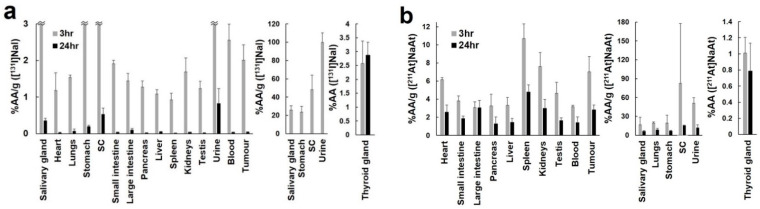
Biodistribution of [^131^I]NaI (**a**) and [^211^At]NaAt (**b**) solution at 3 and 24 h postadministration in the K1-NIS xenograft mice. The percent administered activity (%AA) of the thyroid gland and %AA/g of other organs are expressed as mean ± standard deviation. %AA/g of ^131^I in the salivary gland, stomach, stomach contents, and urine at 3 h and %AA/g of ^211^At in the salivary gland, lungs, stomach, stomach contents and urine are shown separately. SC, stomach contents.

The biodistribution of [^131^I]NaI and [^211^At]NaAt is shown in Figure 3. The thyroid gland, salivary gland, and stomach showed relatively higher uptake of both ^131^I and ^211^At. Although most organs and tumours showed rapid clearance of ^131^I, the uptake of [^131^I]NaI in the thyroid gland was still observed at 24 h, and it was significantly higher than that of [^211^At]NaAt. In other organs and tumours, the uptake of [^211^At]NaAt was significantly higher than that of [^131^I]NaI at 24 h. The absorbed doses of ^131^I and ^211^At in the tumour were 0.115 ± 0.012 and 2.754 ± 0.585 Gy/MBq, respectively.

### 2.3. Changes in Tumour Size and Body Weight in ^131^I Groups

**Figure 4:**
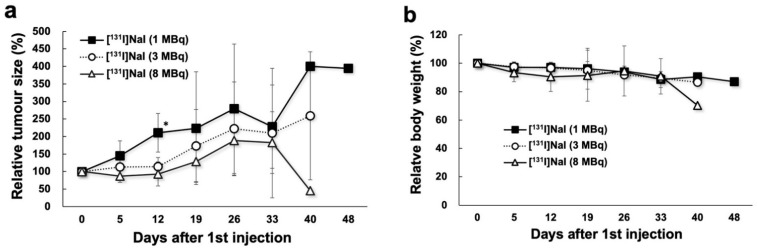
Changes in relative tumour size (**a**) and relative body weight (**b**) in the 1 MBq ^131^I, 3 MBq ^131^I, and 8 MBq ^131^I groups. * *p* < 0.05 between the 1 MBq and 8 MBq groups.

The changes in tumour size and body weight after injection with [^131^I]NaI solution are shown in Figure 4. In the 3 MBq ^131^I group, tumour-suppressive effects were observed immediately after treatment, and 3 MBq of ^131^I was therapeutically more effective than 1 MBq ^131^I. However, the late effect was smaller than the early effect between the 1 MBq and 3 MBq groups. The tumour size of the 8 MBq group showed a trend towards large tumour growth suppression compared with that of the 3 MBq group, although the difference was relatively small. Tumour regrowth was observed within 12 days after injection (1, 4, and 8 MBq).

### 2.4. Changes in Tumour Size and Body Weight in ^211^At Groups

**Figure 5:**
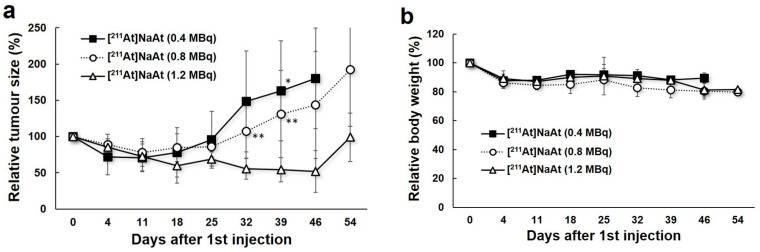
Changes in the relative tumour size (**a**) and relative body weight (**b**) in the 0.4 MBq ^211^At, 0.8 MBq ^211^At, and 1.2 MBq ^211^At group. * *p* < 0.05 between the 0.4 MBq and 1.2 MBq groups; ** *p* < 0.05 between the 0.8 MBq and 1.2 MBq groups.

The results of the administration of the [^211^At]NaAt solution are shown in Figure 5. The regrowth of tumours was suppressed until 18, 25, and 46 days after the administration of 0.4, 0.8, and 1.2 MBq [^211^At]NaAt solution, respectively, in a dose-dependent manner. On day 39, the tumour size of the 1.2 MBq group was significantly smaller than that of the 0.4 MBq group and 0.8 MBq group. Body weight in all three groups decreased slightly during the 2 weeks after the first injection.

## 3. Discussion

The present study showed that [^211^At]NaAt effectively induced more DSBs, with significantly fewer colonies in the in vitro assay, compared with [^131^I]NaI. In tumour xenograft mice, the tumour-growth suppression effects were higher in the ^211^At group than in the ^131^I group despite the lower administered doses in the ^211^At group and shorter physical half-life of ^211^At. 

RAI therapy is now widely used for patients with differentiated thyroid cancer as postsurgical ablation for high-risk patients and for the treatment of recurrence or metastasis. However, a more effective treatment is necessary for patients for whom RAI therapy is insufficient [4,14]. ^211^At is considered to be a potential therapeutic agent for the treatment of differentiated thyroid cancer, and we previously reported the dose-dependent tumour-suppressive effects of [^211^At]NaAt solution [11]. As ^211^At is transported into cells via NIS, similar to ^131^I, its alpha particle emission can achieve a better antitumour effect than ^131^I [10,11]. In addition, outpatient [^211^At]NaAt treatment is possible with minimum radiation exposure to the public and caregivers, although hospitalisation is essential in many countries when administering high dose therapy of [^131^I]NaI [15]. Thus, [^211^At]NaAt therapy may be considered as a better choice for differentiated thyroid cancer with promising therapeutic effects without the need for hospitalisation.

In the present study, we found that ^211^At caused more DSBs in a dose-dependent manner. Previously, we had reported the dose-related induction of DSBs by ^211^At-labeled α-methyl-l-tyrosine in a human pancreatic cancer cell line [16]. Alpha particles emitted by ^211^At have higher LET than beta particles emitted by ^131^I, and the severity and complexity of DNA damage significantly increase with alpha-irradiation [17]. Low LET induces more single strand breaks or isolated DSBs, which can be rejoined 5 h after irradiation [18]. However, high LET irradiation induces more nonrejoining DSBs and clustered lesions, and clustered lesion number and size increase after high LET irradiation, making the repair of DSBs difficult [19,20,21]. The number and rejoining ability of DSBs in cells decreased after alpha-irradiation, and thus the lethality of DSBs increases with alpha-irradiation [22]. 

In addition, we confirmed the lower clonogenicity of [^211^At]NaAt solution in vitro in comparison to [^131^I]NaI, suggesting a lower survival rate of K1-NIS cells treated with the [^211^At]NaAt solution. However, the RBE achieved in the present study is lower than the recommended value for the dosimetry of targeted alpha therapy, that is, 5 [23]. The following three reasons can be considered for this difference: (1) the heterogeneity of the intake of [^211^At]NaAt and [^131^I]NaI into K1-NIS cells, (2) the difference in RBE between in vitro and in vivo experiments, and (3) the uncertainty in the dose estimation. The first reason reduces RBE, particularly that of short-range particles, with high-dose irradiation as discussed in the case of boron neutron capture therapy [24]. This is also the reason for excluding the experimental data for [^211^At]NaAt with the absorbed doses above 5 Gy in the fitting shown in Figure 2b. As for the second reason, we recently revealed that RBE achieved in in vivo experiments tend to be higher than that in in vitro experiments based on the comprehensive analysis of previous observations of skin reaction and dermal cell survival [25]. The last reason is probably the most important, and hence, the accuracy of dosimetry should be improved in the future by performing additional experiments particularly for evaluating the activity concentration with K1-NIS cells during the treatment and cultivation with [^211^At]NaAt or [^131^I]NaI.

Because of the abundant NIS expression in the thyroid gland, salivary gland, and stomach, the relative uptake of ^211^At and ^131^I was similar at 3 h. However, the ^131^I uptake in tissues except the thyroid gland rapidly decreased at 24 h, whereas ^131^I uptake in the thyroid gland was still observed. In previous studies, a short retention time of iodide was also observed in NIS-expressing xenografts. The ^125^I accumulation in the Tc-rNIS xenograft peaked at 90 min but decreased to half at 6 h [26]. Meanwhile, the uptake of ^125^I by organs, except the thyroid gland, ceased at 19 h after administration [27]. Thyroid peroxidase (TPO) is responsible for the effective organification and retention of iodine tracers in thyroid cancer cells and in the normal thyroid [28]. Restoring TPO expression may support the longer retention of radioiodine inside thyroid cancer cells. In contrast, the clearance of ^211^At was slow, and a similar trend was observed in normal rats in a previous study [29]. In addition, Cobb et al. reported that the uptake of ^211^At was higher than ^125^I in the human grafts implanted to mouse (moderately differentiated follicular carcinomas), but lower in the normal mouse thyroid gland [30]. The clearance of ^211^At is slower than that of ^125^I in patient-derived xenografts between 4 and 24 h after administration, which suggested the possibility that ^211^At was retained longer in human thyroid cancers, supporting the findings of the present study. In the interpretation of in vivo results, it is important to consider the difference in tumour uptake and retention between ^211^At and ^131^I, the effect of which might be more than that of the difference between alpha and beta emitters. In addition, it is essential to adjust the absorbed doses to the same level when we compare the real difference between alpha and beta emissions. Further studies are warranted for the precise comparison between ^211^At and ^131^I by comparable adjustment of effective doses as well as the measurement of biodistribution with multiple kinetic points.

The higher number of DSBs caused by alpha-particles and higher uptake of ^211^At in the tumour suggest the possible advantage of ^211^At in the treatment of tumours with NIS expression. Thus, as shown in Figure 4, we observed more effective therapeutic effects in mice administrated 0.4 MBq ^211^At than 3 MBq ^131^I, confirming the stronger tumour-suppressive ability of ^211^At. Meanwhile, the higher absorbed dose of ^211^At in the tumour was also confirmed. The strong treatment response of [^225^Ac]PSMA-617, a targeted alpha therapy, in patients with metastatic prostate cancer who are resistant to [^177^Lu]PSMA-617, also suggests a more beneficial effect of alpha particles than beta particles in clinical application [31].

We primarily targeted the refractory patients with iodine-avid thyroid cancer in our ongoing clinical trial (the investigator-initiated clinical trial) using [^211^At]NaAt, which was initiated in November of 2021 (ClinicalTrials.gov Identifier: NCT05275946). Although most of the refractory patients showed the loss of ^131^I-avidity, some patients still showed enough uptake despite the multiple ^131^I treatments. As ^211^At emits alpha particles with higher LET compared to ^131^I, we expect a therapeutic effect against metastatic lesions even with a low uptake of ^211^At.

In the present study, we determined the dose (Bq) based on the reported values of energy per unit decay (6927 keV/Bq·s for ^211^At and 570 keV/Bq·s for ^131^I) [29]. There is approximately a 10-fold difference between ^211^At and ^131^I in terms of energy per unit decay. Therefore, the maximum dose was 0.1 or 1 MBq for ^211^At and 1 and 10 MBq for ^131^I in the DSB and colony formation assays, with reference doses of 0.4 MBq for ^211^At and 4 MBq for ^131^I in the animal experiment.

Analysis of possible toxicity of [^211^At]NaAt solution in normal organs showed no severe adverse effects with a high administered dose of the [^211^At]NaAt solution (up to 50 MBq/kg) [12,13]. In these studies, thyroid gland ablation, transient bone marrow suppression in the high-dose group (decline in the number of white blood cells and platelet count), and pathological changes in the testis were observed, yet no pathological abnormalities were observed in the other major organs. However, caution should be exercised regarding species difference in biodistribution between mice and humans. Thus, it is necessary to start with a low dose of [^211^At]NaAt solution in the investigator initiated clinical trial. However, low administered doses may result in insufficient tumour suppression or recurrence due to the dose-dependent therapeutic effects of ^211^At. Repeated administrations of [^211^At]NaAt can be considered for future applications in clinical practice.

Severe xerostomia has been reported in patients who received alpha-targeted therapy using [^225^Ac]PSMA-617 [31,32]. Therefore, it is necessary to decrease the potential adverse effects in the clinical application of [^211^At]NaAt. The tandem therapy of [^225^Ac]PSMA-617 and [^177^Lu]PSMA-617 has been shown to enhance efficacy while reducing adverse effects [33], suggesting that the combination of [^211^At]NaAt and [^131^I]NaI may also enhance therapeutic effects with lower toxicity.

This study has some limitations. First, the present study involved a small number of mice due to the limited allowance in our institution for use of radioactivity by legal regulations. Future studies should include a higher number of mice as well as controls for better evaluation with longer periods of observation. Second, we did not compare the adverse effects of the histopathological evaluation between [^211^At]NaAt and [^131^I]NaI administration. In our previous extended single-dose toxicity study of [^211^At]NaAt, we observed a transient mild decrease in white blood cell count at the dose of 20 MBq/kg:0.4 MBq on day 5 after administration, and this decrease was recovered on day 14 even in the high-dose group (50 MBq/kg). In addition, we found no pathological abnormalities in high-uptake organs, such as the salivary gland and stomach, up to 50 MBq/kg [13]. However, there might be accumulated toxicity with the multiple administrations. A detailed comparison of toxicity should be performed in future studies between ^211^At or ^131^I, including histology and blood tests. Furthermore, toxicity in humans will be elucidated during dose escalation in the clinical trial, which has started from a minimal dose. Finally, we did not evaluate the therapeutic effects of the combination of [^211^At]NaAt and [^131^I]NaI, which may have better clinical applications. 

## 4. Materials and Methods

### 4.1. Preparation of [^211^At]NaAt Solution

The ^211^At was acquired from the Research Center for Nuclear Physics at Osaka University and RIKEN through the supply platform of short-lived radioisotopes. The ^211^At was produced according to the ^209^Bi(α, 2n)^211^At reaction and separated from the Bi target using the dry distillation method [9]. The separated ^211^At was dissolved in pure water. Ascorbic acid (used as a reducing agent) and sodium bicarbonate (used as a pH adjuster) were added to the crude ^211^At solution to a final concentration of 1% (*w*/*v*) and 2.1% (*w*/*v*), respectively, at pH 8.0, and the solution was allowed to stand for 1 h at 23 ± 2 °C. The At concentration was 10 MBq/mL. Solutions of [^131^I]NaI were purchased from the Institute of Isotopes Co., Ltd. (Budapest, Hungary).

### 4.2. In Vitro Observation of DSBs of DNA and Colony Formation Assay 

The human papillary thyroid carcinoma cell line K1 was purchased from the European Collection of Authenticated Cell Cultures. NIS expression was induced by the stable transfection of K1 cells with the human *SLC5A5* (NIS) gene clone (OriGene Technologies, Inc., MD, USA). K1 cells were seeded on a 24-well plate, and plasmids were transfected using Lipofectamine2000 (ThermoFisher Sciences, Waltham, MA, USA). After confirming the introduction of the target gene, cloning was performed to obtain clones with strong expression of GFP and NIS proteins, followed by drug selection using G418 (FUJIFILM Wako Chemicals, Osaka, Japan). K1-NIS cells were cultured in a mixed medium of D-MEM (Nacalai Tesque, Inc., Kyoto, Japan), Ham’s F12 (Nacalai Tesque, Inc., Kyoto, Japan), and MCDB 105 (Cell Applications, Inc., San Diego, CA, USA) (2:1:1), supplemented with 10% heat-inactivated foetal bovine serum (Corning, New York, NY, USA), 2 mM glutamine (Nacalai Tesque, Kyoto, Japan), and 1% penicillin–streptomycin solution (Nacalai Tesque, Kyoto, Japan). 

For the measurement of DSBs, K1-NIS cells were seeded in an eight-well chamber slide at a density of 3 × 10^5^ cells/mL. After 2 days of incubation, the cells were treated with 10 μL medium/well as control group; 1, 3, 10, 30, and 100 kBq [^211^At]NaAt solution/well as ^211^At groups; and 10, 30, 100, 300, and 1,000 kBq [^131^I]NaI solution/well as ^131^I groups for 20 min. The volume of the solution was approximately 325 μL/well during the treatment. After washing with phosphate-buffered saline (-), the cells were stained using the HCS DNA Damage Kit (Thermo Fisher Scientific, Inc., Waltham, MA, USA). Fluorescence signals were observed using a fluorescence microscope (BZ-9000; Keyence Corporation, Osaka, Japan). The percentage of DSB induction was calculated using ImageJ software and compared between the groups. Cells of interest were selected, and the areas of nuclear morphology (Hoechst 33342) and DNA damage (pH2AX antibody) were measured. Percentage of DSB induction (DSBs%) = the area of DNA damage/nuclear morphology × 100.

K1-NIS cells were seeded in six-well plates until 70%–80% confluency and were detached for the colony formation assay using trypsin-EDTA solutions (Nacalai Tesque, Kyoto, Japan). To minimize the damage to cell membrane protein by trypsin, we completed the cell detachment procedure in a short time. The cells in each well were treated with 5, 10, 20, 50, 100, 200, 500, and 1000 kBq [^211^At]NaAt solution and 0.5, 1, 2, 3, 4, 5, 8, and 10 MBq [^131^I]NaI solution. The volume of solution was 2 mL. The doses continuously covered 0%–100% values of % colony-forming units. After 1 h of treatment at 37 °C in a humidified atmosphere of 5% CO_2_, the cells were counted and seeded in fresh medium in six-well plates at 1000 cells/well. After 14 days of incubation, the cells were fixed and stained with a crystal violet solution [34]. Cells were viewed and counted under a microscope (BZ-X810, Keyence Corporation, Osaka, Japan).

### 4.3. Dosimetry of In Vitro Observation

The absorbed doses in K1-NIS cells during the treatment, *D*_T_, were estimated as

(1) $$D_{T}=\frac{1-e^{-\lambda_{p}t_{T}}}{\lambda_{p}}\left(A_{c}V_{c}S_{c\to c}+A_{s}V_{s}S_{s\to c}\right),$$

where, *t*_T_ is the treatment time (i.e., 20 min for DSB observation and 1 h for the colony formation assay), $\lambda_{p}$ is the physical decay constant of the radioisotopes (RI), *A*_c_ and *A*_s_ are their activity concentrations, and *V*_c_ and *V_s_*. are the total volumes of the cells and solution, respectively. *S*_c__→__c_ and *S*_s__→__c_ indicate the absorbed doses in the cells due to a single decay of RI located in the cells and solution, respectively, the so-called S-value. For estimating the S values, we performed Monte Carlo radiation transport simulation using the Particle and Heavy Ion Transport Code System (PHITS) [35]. In the simulation, dish, cells, and solution were represented by coaxis cylinders of diameter 11.1 mm (or 34.6 mm) and heights 1 mm, 3 μm, and 3.3 mm (or 2.1 mm), respectively, where the values in parentheses are for reproducing the treatment of the colony formation assay. The α, β, and γ-rays were generated from either cell or solution region, and their energy spectra were determined using the RI source function in PHITS, considering the contributions from the daughter nuclides. The calculated *S*_c__→__c_ and *S*_s__→__c_ were 33.5 and 0.247 nGy.(Bq.s)^−1^ for [^211^At]NaAt, and 355 and 9.74 pGy.(Bq.s)^−1^ for [^131^I]NaI, respectively. For estimating *A*_c_ and *A*_s_, we assumed that 11.6% of [^211^At]NaAt and 32.5% of [^131^I]NaI were accumulated in K1-NIS cells from the cellular uptake analysis after 30 min of incubation with [^211^At]NaAt or [^131^I]NaI.

In the treatment for the colony formation assay with the administered dose of 1 MBq, the calculated *D*_T_ was 7.05 and 0.219 Gy for [^211^At]NaAt and [^131^I]NaI, respectively. The statistical uncertainties of the calculated results were negligibly small—less than a few percent. However, their systematic uncertainties are rather large, a factor of 2 at the maximum, due to the rough estimations of the cell thickness and the activity ratios in the cells and solution. For example, the calculated dose would decrease approximately 15% if the thickness of cells is 5 μm instead of 3 μm.

### 4.4. Preparation of Animals 

Male severe combined immune-deficient mice were purchased from Charles River Japan, Inc. (Atsugi, Japan), housed under a 12 h light/12 h dark cycle, and allowed free access to food and water. The mice were injected with K1-NIS cells (1 × 10^7^ cells) in 0.2 mL of culture medium and Matrigel (1:1; BD Biosciences, Franklin Lakes, NJ, USA) into the right flank. The tumour size was approximately 10 mm in diameter, with a growth phase of 4 weeks, before the administration of [^131^I]NaI or [^211^At]NaAt solution.

### 4.5. Biodistribution of [^131^I]NaI and [^211^At]NaAt in Mice

K1-NIS xenograft mice (body weight = 20.59 ± 3.82 g) were used to evaluate biodistribution after the administration of [^131^I]NaI solution (1.20 ± 0.040 MBq, n = 6) or [^211^At]NaAt solution (0.12 ± 0.004 MBq, n = 6). The thyroid gland, salivary gland, heart, lungs, stomach, stomach content, small intestine, large intestine, pancreas, liver, spleen, kidneys, testis, urine, blood, and tumour were removed and weighed for biodistribution evaluation after euthanasia by deep anaesthesia via inhalation of isoflurane at 3 and 24 h. The radioactivity was measured using a gamma counter (AccuFLEX γ7000, Aloka, Tokyo, Japan). Equivalent doses in the tumour were calculated according to a previous study [27]. The absorbed fraction was set to 1.0 for both ^131^I and ^211^At.

### 4.6. Therapy with [^131^I]NaI and [^211^At]NaAt Solutions

Mice injected with [^131^I]NaI solution through the tail vein were divided into the following three groups, according to the injected dose: 1 MBq ^131^I group (1.00 ± 0.19 MBq, n = 4), 3 MBq ^131^I group (3.44 ± 0.34 MBq, n = 4), and 8 MBq ^131^I group (8.15 ± 0.27 MBq, n = 4, multiple administrations in duplicate of 4 MBq at an interval of 5 days). Mice injected with [^211^At]NaAt solution through the tail vein were divided into the following three groups, according to the injection dose: 0.4 MBq ^211^At group (0.38 ± 0.06 MBq, n = 7), 0.8 MBq ^211^At group (0.82 ± 0.06 MBq, n = 9, multiple administrations in duplicate at an interval of 11–16 days), 1.2 MBq ^211^At group (1.20 ± 0.04 MBq, n = 4 multiple administrations in triplicate at an interval of 17 days). Tumour size and body weight were also measured.

### 4.7. Statistical Analysis

Results are expressed as mean ± standard deviation. Comparisons between groups were performed using an unpaired *t*-test in Microsoft Excel (version 2016). For multiple comparisons among three groups, Bonferroni correction was performed. Differences were considered statistically significant at *p* < 0.05.

## 5. Conclusions

In this study, ^211^At showed effective DSB induction with higher cellular toxicity, and the administration of [^211^At]NaAt was more effective in a NIS-expressing thyroid cancer model than the administration of [^131^I]NaI. The results suggest that [^211^At]NaAt therapy is a promising option for patients with iodine-avid thyroid cancer refractory to [^131^I]NaI treatment.

## Data Availability

All data are included in this published article.
